# Fatal *Streptococcus equi* subsp. *ruminatorum* Infection in a Man

**DOI:** 10.3201/eid1312.070109

**Published:** 2007-12

**Authors:** Hélène Marchandin, Estelle Jumas-Bilak, Abderrahmane Boumzebra, Delphine Vidal, Olivier Jonquet, Philippe Corne

**Affiliations:** * Centre Hospitalier Universitaire de Montpellier, Montpellier, France; † Unité de Formation et de Recherche des Sciences Pharmaceutiques, Montpellier, France; ‡ Institut de Recherche pour le Développement, Montpellier, France

**Keywords:** *Streptococcus equi* subspecies *ruminatorum*, HIV, 16S ribosomal RNA gene, zoonosis, meningitis, bacteremia, letter

**To the Editor:** *Streptococcus equi* belongs to the pyogenic group of streptococci and to group C of the Lancefield classification. It consists of 3 subspecies of zoonotic agents rarely reported as human pathogens (*1*,*2*): *S. equi* subsp. *equi*, *S. equi* subsp. *zooepidemicus*, and *S. equi* subsp. *ruminatorum*. We report here a case of human infection caused by *S. equi* subsp. *ruminatorum*. (*3*).

A 53-year-old man was admitted to an intensive care unit of our hospital (University Teaching Hospital, Montpellier, France) on April 28, 2006, with a high fever and in a comatose state. The day before, he had experienced headache and neck pain. He had been infected with HIV for 9 years but had not had an opportunistic infection. His ongoing HIV treatment consisted of ritonavir, lopinavir, abacavir, lamivudine, and co-trimoxazole; 3 weeks before admission, his blood CD4+ T-cell count was 133/μL, and viral load was 118,000 copies/mL. At the time of admission, his body temperature was 38.9°C, heart rate was 105 beats/min, and blood pressure was 55/35 mmHg. He exhibited a fixed pupil in 1 eye, neck stiffness, and was nonresponsive. He had bilateral pulmonary infiltrates and severe hypoxemia. Treatment consisted of mechanical ventilation, fluid therapy, and norepinephrine. Laboratory investigations found the following: leukocyte count 9,600/mm^3^ with 90% neutrophils, hemoglobin level 9.0 g/dL, platelet count 32,000/mm^3^, C-reactive protein value 159 mg/L, and blood lactate concentration 3.2 mmol/L. Computed tomographic scanning of the brain showed no hemorrhage or edema. Lumbar puncture produced turbid cerebrospinal fluid (CSF) with 300 leukocytes/mm^3^ (95% neutrophils), protein 5.6 g/L, glucose <0.1 mmol/L, and gram-positive cocci. Three sets of aerobic-anaerobic blood cultures and bronchial aspirates were sampled, and intravenous treatment with dexamethasone (10 mg/6 h/day), cefotaxime (2 g/4 h/day), and vancomycin (30 mg/kg/day) was initiated. On day 2, the hemodynamic state was stabilized, but brain death occurred.

All sets of aero-anaerobic blood cultures, CSF, and bronchial aspirate fluid yielded the growth of a catalase-negative, β-hemolytic, gram-positive cocci belonging to the Lancefield group C of streptococci. Antimicrobial susceptibility testing showed a bacterium fully susceptible to antibiotics tested. MICs of penicillin, amoxicillin, and cefotaxime were 0.047, 0.125, and 0.125 mg/L, respectively. The isolates were identified as *S. equi* by using the Vitek2 system, rapid ID32 STREP, and API 20 STREP strips (bioMérieux, Marcy l’Etoile, France), but phenotype was inconclusive for subspecies identification. The strains were identified as *S. equi* subsp. *zooepidemicus* by Vitek2, but aesculin was not hydrolyzed, and D-ribose fermentation was noted, as previously described for *S. equi* subsp. *ruminatorum*. 16S rRNA gene–based identification was performed as previously described (*4*) on strain ADV 6048.06 from blood. The 1,396-bp sequence (GenBank accession no. EF362949) was compared with databases by using the BLAST program (*5*); the sequence differed by only 1 nucleotide position (>99.9% identity) from the sequence of *S. equi* subsp. *ruminatorum* CECT 5772^T^. Other primarily related sequences were from *S. equi* subsp. *ruminatorum* strains of animal origin (99.5%–99.9% identity) and from *S. equi* subsp. *zooepidemicus*, (98.7% identity). Phylogenetic trees clustered the clinical isolate with *S. equi* subsp. *ruminatorum* strains to form a robust lineage, well separated from other strains of *S. equi* and supported by a high bootstrap value (Figure).

**Figure:**
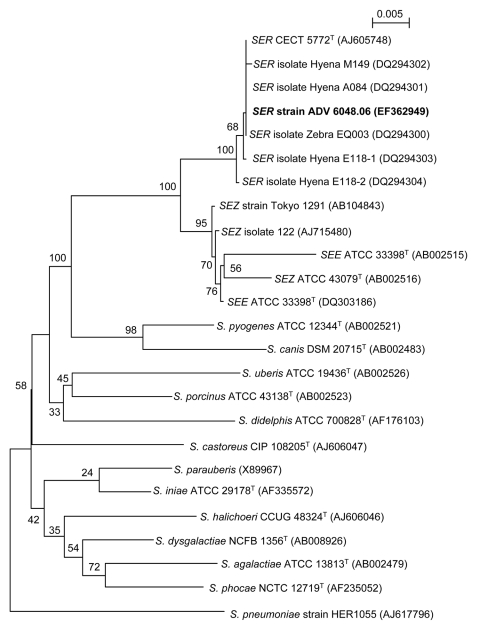
Neighbor-joining tree showing the phylogenetic placement of strain ADV 6048.06 (**boldface**) among members of the *Streptococcus equi* species in the pyogenic group of streptococci. Twenty-three 16S rRNA gene sequences selected from the GenBank database were aligned with that of strain ADV 6048.06 by using ClustalX 1.83 (available from http://bips.u-strasbg.fr/fr/documentation/ClustalX). Alignment of 1,263 bp was used to reconstruct phylogenies by using PHYLIP v3.66 package (http://evolution.genetics.washington.edu/phylip.html). The neighbor-joining tree was constructed with a distance matrix calculated with F84 model. Numbers given at the nodes are bootstrap values estimated with 100 replicates. *S. pneumoniae* is used as outgroup organism. Accession numbers are indicated in brackets. The scale bar indicates 0.005 substitutions per nucleotide position. Maximum likelihood and parsimony trees were globally congruent with the distance tree and confirmed the placement of the strain ADV 6048.06 in the *S. equi* subspecies *ruminatorum* (SE*R*) lineage. SE*Z*, *S. equi* subspecies *zooepidemicus.*

*S. equi* subsp. *equi* and *S. equi* subsp. *zooepidemicus* are zoonotic agents implicated in diverse animal infections such as strangles, mastitis, abscesses, wounds, and respiratory and uterine infections. Human infections caused by *S. equi* subsp. *equi*, and *S. equi* subsp. *zooepidemicus* included outbreaks of foodborne diseases (*6*,*7*), meningitis, septicemia, arthritis, pneumonia, glomerulonephritis, and streptococcal toxic shock syndrome, in both immunocompromised and immunocompetent patients (*1*,*2*,*8*,*9*). *S. equi* subsp. *ruminatorum* was described in 2004 in domestic sheep and goats with mastitis (*3*). More recently, it was isolated during severe infections in spotted hyenas and zebras (*10*). No human isolate has been reported to date. Moreover, none of the 3 subspecies of *S. equi* has been isolated from HIV-infected patients. The current case underlines the conclusion that molecular identification of *S. equi* subsp. *ruminatorum* is essential. *S. equi* subsp. *ruminatorum* could have been underestimated due to its potential misidentification as *S. equi* subsp. *zooepidemicus* by phenotypic tools. Despite the rare occurrence of group C streptococci in human infections, a high death rate is reported for invasive infections (*7*–*9*). *S. equi* subsp. *zooepidemicus* produce superantigen exotoxin that may have been implicated in the pathogenesis of fatal infection (*2*); *S. equi* subsp. *ruminatorum* should also be investigated for potential virulence factors for humans.

Epidemiologic investigations were unsuccessful in tracing the patient’s infection to an animal source. The respiratory tract, from which *S. equi* subsp. *ruminatorum* was recovered in pure culture, could be considered the most probable portal of entry.

The mode of *S. equi* subsp. *ruminatorum* transmission to humans remains unknown. More information is needed on its reservoirs, but they likely resemble those of *S. equi* subsp. *equi*, and *S. equi* subsp. *zooepidemicus* (*2*,*6*,*7*). Prevention of human infections due to *S. equi* should include frequent microbiologic sampling of lactating animals and control measures for unpasteurized dairy products (*7*). Better characterization of underlying conditions that increase risk of invasive *S. equi* infections is also needed. This knowledge could help define high-risk groups of persons and could lead to generation of specific preventive recommendations.
